# A nomogram for *P *values

**DOI:** 10.1186/1471-2288-10-21

**Published:** 2010-03-16

**Authors:** Leonhard Held

**Affiliations:** 1 Biostatistics Unit, Institute of Social and Preventive Medicine, University of Zurich, Hirschengraben 84, 8001 Zurich, Switzerland

## Abstract

**Background:**

*P *values are the most commonly used tool to measure evidence against a hypothesis. Several attempts have been made to transform *P *values to minimum Bayes factors and minimum posterior probabilities of the hypothesis under consideration. However, the acceptance of such calibrations in clinical fields is low due to inexperience in interpreting Bayes factors and the need to specify a prior probability to derive a lower bound on the posterior probability.

**Methods:**

I propose a graphical approach which easily translates any prior probability and *P *value to minimum posterior probabilities. The approach allows to visually inspect the dependence of the minimum posterior probability on the prior probability of the null hypothesis. Likewise, the tool can be used to read off, for fixed posterior probability, the maximum prior probability compatible with a given *P *value. The maximum *P *value compatible with a given prior and posterior probability is also available.

**Results:**

Use of the nomogram is illustrated based on results from a randomized trial for lung cancer patients comparing a new radiotherapy technique with conventional radiotherapy.

**Conclusion:**

The graphical device proposed in this paper will enhance the understanding of *P *values as measures of evidence among non-specialists.

## Background

*P *values are the most commonly used tool to measure evidence against a hypothesis [1]. The *P *value is defined as the probability, under the assumption of no effect (the null hypothesis *H*_0_), of obtaining a result equal to or more extreme than what was actually observed. The complexity of this definition has led to widespread misinterpretations and criticisms [2-5]. Indeed, *P *values are often misinterpreted (a) as the probability of obtaining the observed data under the assumption of no real effect, (b) as an "observed" type-I error rate, (c) as the false discovery rate, i.e. the probability that a significant finding is "false positive", and (d) as the (posterior) probability of the null hypothesis [6].

The latter misinterpretation has given rise to interesting work on the connection between *P *values and (posterior) probabilities of the null hypothesis. Within a Bayesian framework, the posterior probability is a function of the prior probability and the so-called Bayes factor, which summarizes the evidence against the null hypothesis.

Several attempts have been made to transform *P *values to lower bounds on the Bayes factor and the resulting posterior probability of the null hypothesis [7-11]. In this context Bayes factors are usually oriented as *P *values such that smaller values provide stronger evidence against the null hypothesis. These techniques calibrate *P *values such that an interpretation as minimum Bayes factor or minimum posterior probability is justified. Although the different approaches do not result in identical calibration scales, a universal finding is that the evidence against a simple null hypothesis is by far not as strong as the *P *value might suggest.

However, the acceptance of calibrated *P *values in clinical fields is low. Minimum Bayes factors have the advantage that they do not depend on the prior probability of the null hypothesis [9], but their interpretation requires an intuitive understanding of odds, similar to likelihood ratios in diagnostic studies [12]. Clinicians, however, prefer to think in terms of probabilities. The calculation of the minimum posterior probability, on the other hand, requires to decide on a prior probability of the null hypothesis. Fixing a prior probability may be difficult for the clinician, who would perhaps prefer to investigate - for a given *P *value - the dependence of the (minimum) posterior probability of the null hypothesis on the prior probability.

In this paper I propose a graphical approach, which easily translates any prior probability and *P *value into minimum posterior probabilities. Likewise, the tool can be used to derive, for fixed posterior probability, the maximum prior probability compatible with a given *P *value. The maximum *P *value in accordance with a given prior and posterior probability can be also read off. The approach is inspired by the Fagan nomogram [13] used to derive the post-test probability in diagnostic tests [12]. It will enhance the understanding and facilitate the interpretation of *P *values as measures of evidence against the null hypothesis among non-specialists.

## Methods

### Calibration of *P *values

In a seminal paper, Edwards, Lindman and Savage [7] (ELS) studied the relationship between *P *values and minimum Bayes factors in several settings. Of particular interest is the case where a test statistic is normal distributed with unknown mean *μ*. A simple null hypothesis *H*_0 _corresponds to a particular mean value *μ *= *μ*_0_. Calculation of the Bayes factor requires fixing a prior density for *μ *under the alternative hypothesis *H*_1_: *μ *≠ *μ*_0_.

This scenario reflects, at least approximately, many of the statistical procedures found in medical journals.

The minimum Bayes factor turns out to be

here *z *is the *z*-value, i.e. the test statistic which has given rise to the observed *P *value. This lower bound can be derived using the fact that the Bayes factor is minimized if the alternative hypothesis has all its prior density at one particular value of *μ *supported most by the data (the Maximum Likelihood estimate). Because this point is always on one side of the null hypothesis, ELS suggested to use a *z*-value based on a one-tailed rather than a two-tailed significance test. A two-tailed test, which leads to slightly larger values of *z *and to slightly smaller values of BF has also been suggested [9].

For a fixed prior probability *q*, say, of the null hypothesis, the minimum Bayes factor BF can easily be transformed into a lower bound on the posterior probability of the null hypothesis based on Bayes' theorem:(1)

The first row in Table 1 gives this lower bound for *q *= 50% and *P *values of 0.05, 0.01 and 0.001, respectively, using the ELS approach. A striking feature is that the lower bound for the posterior probability is considerably larger than the corresponding *P *value.

**Table 1:** Lower bounds on the posterior probability of the null hypothesis for different *P *values and equal prior probabilities of null and alternative hypothesis (*q *= 50%).

	*P *value		
Method	0.05	0.01	0.001
Edwards, Lindman, and Savage (1963)	20.5%	6.3%	0.8%
Berger and Sellke (1987, Scenario 1)	22.7%	6.8%	0.9%
Sellke, Bayarri, and Berger (2001)	28.9%	11.1%	1.8%
Berger and Sellke (1987, Scenario 2)	29.0%	10.9%	1.8%
Berger and Sellke (1987, Scenario 3)	32.1%	13.3%	2.4%

The ELS approach has been refined by Berger and Sellke [8] (BS). They derived lower bounds for the Bayes factor under more realistic families of prior distributions for *μ *under the alternative hypothesis. In particular, they considered (1) symmetric prior distributions, (2) unimodal and symmetric prior distributions, and (3) normal prior distributions, all centered at *μ*_0_. As one would expect, the corresponding lower bounds on the posterior probability of *H*_0 _increase with increasing restrictions on the prior family for *μ*, as can be seen in Table 1.

Perhaps the simplest and most intuitive calibration has been suggested by Sellke, Bayarri and Berger [10] (SBB). They use the fact that a *P *value is (under suitable regularity conditions) uniformly distributed if *H*_0 _is true. Under the alternative hypothesis smaller *P *values are more likely than larger *P *values, i.e. the density of the *P *value is monotonically decreasing. A flexible class of decreasing densities on the unit interval is provided by specific beta densities with one unknown parameter. The minimum Bayes factor is then

Here *p *is the observed *P *value and *e *= exp(1) ≈ 2.718 is Euler's constant. The resulting lower bounds on the posterior probability of the null hypothesis are very similar to those obtained using the BS approach with a unimodal prior density for *μ*, as can be seen from Table 1. Note that the SBB bounds hold in a more general setting without the assumption of a beta distributed *P *value under the alternative hypothesis [10]. More recently, minimum Bayes factors for *χ*^2^-distributed test statistics have been studied [11]. Such test statistics have an additional parameter, the degrees-of-freedom *ν*, which depends on the specific type of test applied. The following lower bound on the Bayes factor has been derived:

Here, *x *is the value of the *χ*^2^-test statistic which has given rise to the observed *P *value. It can be easily shown that BF decreases with increasing degrees-of-freedom. Perhaps more interestingly, BF is equal to the BS lower bound for normal priors for *ν *= 1, equals the SBB lower bound for *ν *= 2, and is equal to the ELS lower bound for *ν *→ ∞. This illustrates that the range of lower bounds on the posterior probability given in Table 1 reflects a large variety of different tests and scenarios.

### A nomogram for *P *values

The apparent complexity of the formulae presented in the previous section may be one of the reasons why the proposed calibration of *P *values has not entered routine scientific research. I therefore suggest to adapt a graphical device, originally developed for diagnostic tests [13], to the setting outlined above. The original Fagan nomogram allows to visually determine the post-test probability for a given pre-test probability and a likelihood ratio in a diagnostic test framework [12]. The likelihood ratio is a function of sensitivity, specificity and the actual result of the diagnostic test considered. The likelihood ratio is a specific form of a Bayes factor where both hypotheses under consideration (either the patient has the disease or not) are simple and no additional prior assumptions have to be made.

**Figure 1:**
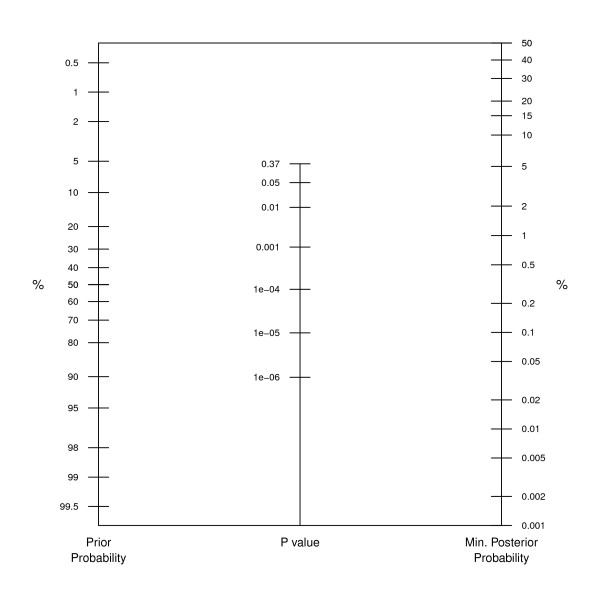
**A nomogram for *P *values**. The prior probability for the null hypothesis is located on the first axis, the observed *P *value on the second axis, and the minimum posterior probability on the third axis.

The proposed graphical device is shown in Figure 1. The prior probability for the null hypothesis is located on the first axis and joined to the observed *P *value on the second axis. The minimum posterior probability is then read off the third axis. The *P *value scaling on the second axis is based on the SBB calibration. Of course, any other of the calibrations discussed in the previous section could have been used, but the SBB approach seems particularly suitable since it is not designed for a specific test statistic (normal or *χ*^2^) but is derived in a more general setting.

Note that there are some notable differences compared with the original Fagan nomogram. First, the likelihood ratio is replaced with the *P *value. Secondly, only *P *values smaller than 1/*e *≈ 0.37 are considered since BF is unity for larger *P *values, where there is lack of evidence against the null hypothesis. Therefore the prior probability scale on the left-hand side of the plot is not identical to the posterior probability scale on the right-hand side of the plot. This reflects the fact that *P *values are asymmetric measures of evidence, they quantify the evidence against the null hypothesis, but they do not quantify the evidence in favour of the null hypothesis. This is different in the Fagan nomogram, where likelihood ratios can be both larger and smaller than unity. Finally, the third axis gives not an exact value for the posterior probability of the null hypothesis but only the minimum posterior probability.

## Results

The proposed nomogram can be used in three different ways, as will be illustrated by the following example. In 1986 a new radiotherapy technique called CHART was introduced. Promising pilot studies led the UK Medical Research Council to instigate a large randomized trial for lung cancer patients. The objective of the study was to estimate the change in survival when given CHART compared with conventional radiotherapy.

**Figure 2:**
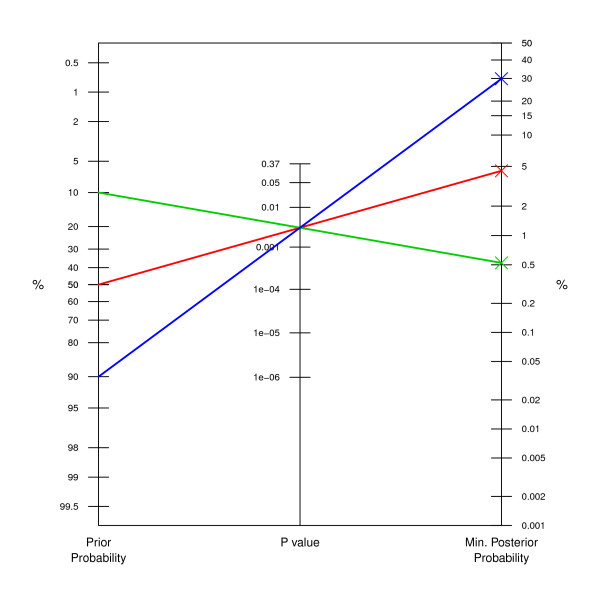
**Application to lung cancer CHART trial**. For a *P *value of 0.3% (0.003), the lower bound on the posterior probability can be read off the third axis for a *q *= 10% (green line), *q *= 50% (red line), and *q *= 90% (blue line) prior probability.

Before the trial a *q *= 10% prior chance that CHART would offer no survival benefit at all was elicited from 11 clinicians [5]. At the end of the trial a clinically important and statistically significant difference in survival was found (9% improvement in 2 year survival, 95% CI: 3-15%, Two-sided *P *value = 0.3%, i.e. 0.003) [14]. We can now easily read off the lower bound of around 0.5% for the posterior probability of the null hypothesis (green line in Figure 2).

Due to the relatively small prior probability, the minimum posterior probability of the null hypothesis is in this example numerically quite close to the *P *value. This will be different for larger prior probabilities. For example, for *q *= 50% we obtain a minimum posterior probability of no survival benefit of around 4.5% (red line). For *q *= 90% the minimum posterior probability is 29.9% (blue line).

**Figure 3:**
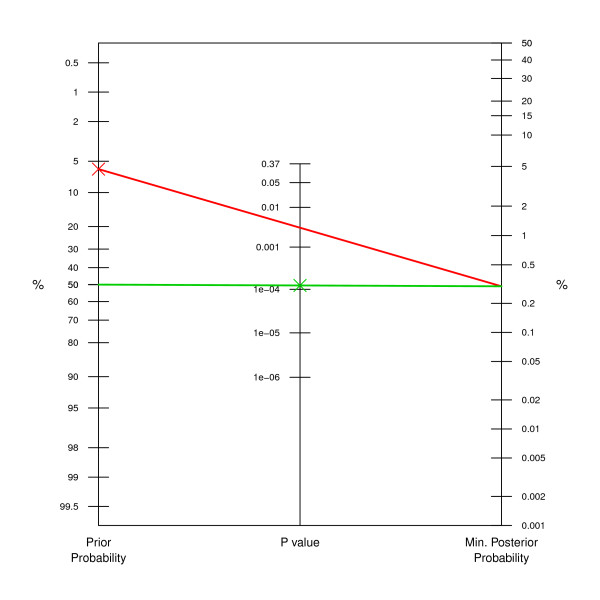
**Application to lung cancer CHART trial**. The prior probability must be 6% or smaller to obtain a lower bound of 0.3% on the posterior probability (red line). The green line indicates, that we need a *P *value of 0.014% (0.00014) or smaller to reduce the probability of the null hypothesis from *q *= 50% *a priori *to 0.3% *a posteriori*.

There are two other ways how to use the nomogram, solving for either the prior probability or the *P *value. For example, to obtain a posterior probability of 0.3% with a *P *value of 0.3%, the prior probability must be 6% or smaller, as can be read off from the red line in Figure 3. Alternatively, one might be interested in the maximum *P *value that is compatible with a reduction of the probability of the null hypothesis from 50% *a priori *to 0.3% *a posteriori*, say. Figure 3 indicates (green line) that we need a *P *value of 0.014% (0.00014) or smaller to achieve this, more than one order of magnitude smaller than the targeted posterior probability of 0.3%.

## Discussion

The Fagan nomogram [12] is widely used in the context of diagnostic tests and I hope that the proposed nomogram for *P *values will reach similar popularity. It visually transforms *P *values to minimum posterior probabilities of the null hypothesis and thus avoids complicated calculations. Sensitivity with respect to prior assumptions can be studied graphically. In addition, for fixed posterior probability, the maximum prior probability compatible with a given *P *value can be read off. The maximum *P *value compatible with a given prior and posterior probability is also available.

**Figure 4:**
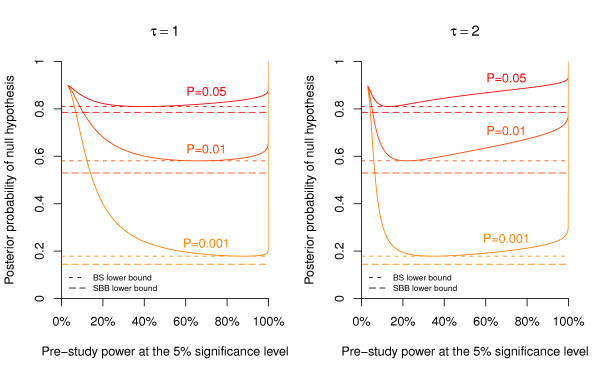
**Dependence of the posterior probability on study power**. Posterior probability of the null hypothesis plotted against the (pre-study) power at the 5% significance level for *P *= 0.05, 0.01, and 0.001 and a prior probability of *q *= 90%. The calculation is based on a normal prior with standard deviation *τ *= 1 (left plot) and *τ *= 2 (right plot) under the alternative, assuming that one unit corresponds to the minimum clinically important difference. The dashed lines indicate the minimum posterior probability as obtained from the BS (short dashed) and SBB (long dashed) approach, respectively.

As emphasized in Spiegelhalter *et al. *[[5], p. 130-133], the actual posterior probability of the null hypothesis will also depend on the power (*i.e. *sample size) of the study. However, Hooper [15] has recently shown that the evidence against the null hypothesis provided by a precise *P *value does not strongly depend on power over the range of study sizes that are commonly encountered in clinical and epidemiological research. For illustration, we reproduce in our Figure 4 the top panel of Figure 3 from Hooper [15], which gives the posterior probability of the null hypothesis plotted against the (pre-study) power at the 5% significance level for *P *= 0.05, 0.01, and 0.001. The calculation is based on a normal prior with mean *μ*_0 _and standard deviation *τ *= 1 (left plot) and *τ *= 2 (right plot) under the alternative (assuming that one unit corresponds to the minimum clinically important difference). This corresponds to Scenario 3 from Berger & Sellke [8]. We have added the corresponding BS lower bound (short dashed) on the posterior probability in Figure 4. The actual posterior probability is quite close to this minimum for all powers typically encountered in clinical research, say between 40% and 95%. This holds both for *τ *= 1 (left plot in Figure 4) and *τ *= 2 (right plot). Only for very small or very large studies the posterior probability is considerably greater than the BS lower bound. The SBB bound, given by the long dashed line, is more conservative and hence slightly lower than the BS lower bound.

In this paper I have adopted a Bayesian approach to calculate a lower bound on the posterior probability of the null hypothesis, derived from a prior probability and a precise *P *value. Even Cox [[16], p. 83] agrees that "conclusions expressed in terms of probability are on the face of it more powerful than those expressed indirectly via confidence intervals and *P *values. Further, in principle at least, they allow the inclusion of a richer pool of [prior] information." However, Cox feels that "conclusions derived from the frequentist approach are more immediately secure than those derived from most Bayesian analysis" because [prior] "information is typically more fragile or even nebulous as compared with that typically derived more directly from the data under analysis". On the other hand, Goodman [1,3,6,9] argues that the misunderstanding and misuse of *P *values is so widespread that new tools are needed to properly convey the strength of evidence provided by research data. The nomogram proposed in this paper is such a tool and is particularly useful to study sensitivity to the prior probability of the null hypothesis, as illustrated in Figure 2. Combined with a precise *P *value we obtain a range of plausible values for the posterior probability of the null hypothesis, which is far easier to interpret than the *P *value itself.

## Conclusions

The graphical device proposed in this paper enhances the understanding and facilitates the interpretation of *P *values as measures of evidence against the null hypothesis among non-specialists. For study sizes typically encountered in clinical and epidemiological research, the posterior probability of the null hypothesis will be quite close to the lower bound provided by the nomogram. We are currently preparing a JAVA applet at http://www.biostat.uzh.ch/static/pnomogram which allows to interactively use the proposed nomogram on the internet.

## Statement of competing interests

I declare that I have no competing interests.

## Pre-publication history

The pre-publication history for this paper can be accessed here:

http://www.biomedcentral.com/1471-2288/10/21/prepub
